# Platelet-derived growth factor alpha and beta receptors have overlapping functional activities towards fibroblasts

**DOI:** 10.1186/1755-1536-6-10

**Published:** 2013-05-10

**Authors:** Johanna Donovan, Xu Shiwen, Jill Norman, David Abraham

**Affiliations:** 1 Centre for Rheumatology and Connective Tissue Diseases and Division of Medicine, UCL Medical School, Royal Free Campus, London, UK; 2 Centre for Nephrology, Division of Medicine, UCL Medical School, Royal Free Campus, London, UK

## Abstract

**Background:**

Platelet-derived growth factor (PDGF) signalling is essential for many key cellular processes in mesenchymal cells. As there is redundancy in signalling between the five PDGF ligand isoforms and three PDGF receptor isoforms, and deletion of either of the receptors *in vivo* produces an embryonic lethal phenotype, it is not know which ligand and receptor combinations mediate specific cellular functions. Fibroblasts are key mediators in wound healing and tissues repair. Recent clinical trials using broad spectrum tyrosine kinase inhibitors in fibrotic diseases have highlighted the need to further examine the specific cellular roles each of the tyrosine kinases plays in fibrotic processes. In this study, we used PDGFR-specific neutralising antibodies to dissect out receptor-specific signalling events in fibroblasts *in vitro*, to further understand key cellular processes involved in wound healing and tissue repair.

**Results:**

Neutralising antibodies against PDGFRs were shown to block signalling through PDGFRα and PDGFRβ receptors, reduce human PDGF-AA and PDGF-BB-induced collagen gel remodelling in dermal fibroblasts, and reduce migration stimulated by all PDGF ligands in human dermal and lung fibroblasts.

**Conclusions:**

PDGFRα and PDGFRβ neutralising antibodies can be a useful tool in studying PDGFR isoform-specific cellular events.

## Background

**Figure 1:**
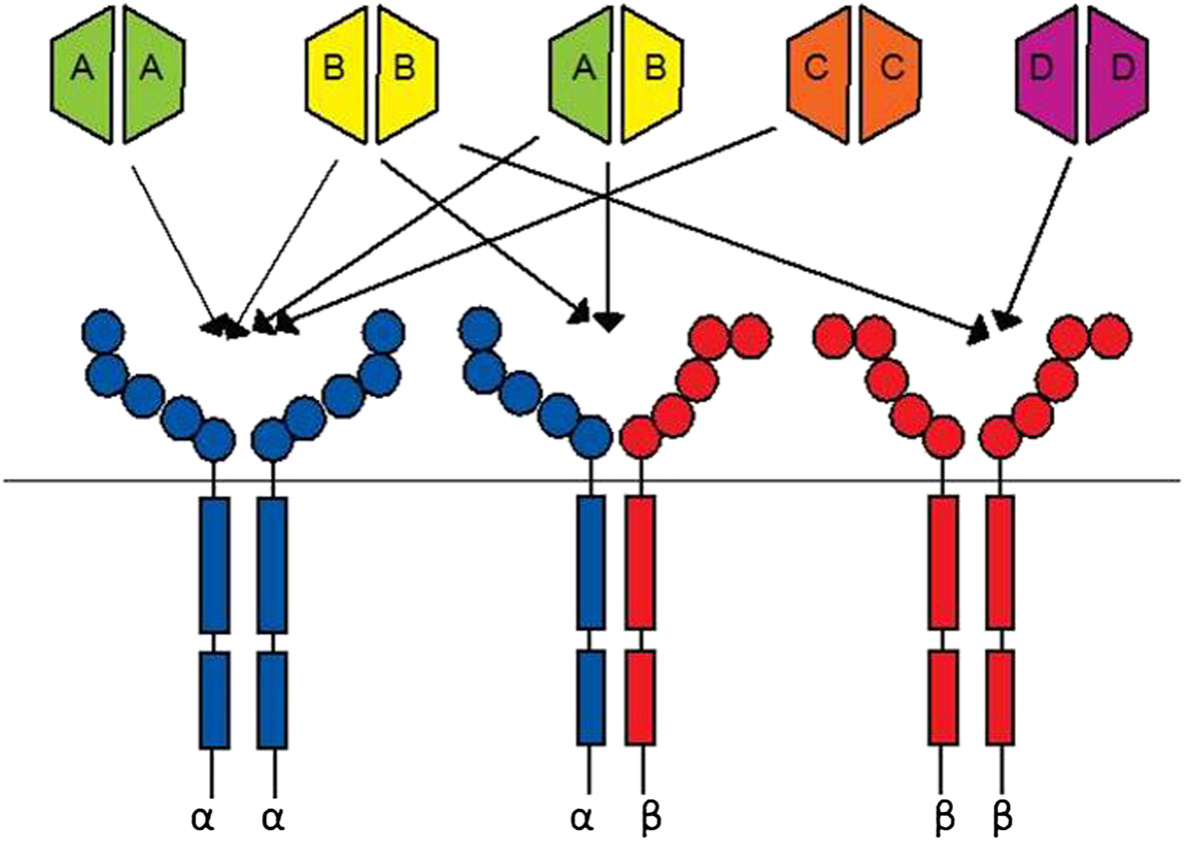
**Schematic diagram showing PDGFR-PDGF interactions *in vitro: *PDGF ligand dimers bind either PDGFRα or PDGFRβ homodimers or the α/β heterodimer.***Arrows* show proven *in vitro* ligand-receptor interactions. Each PDGF receptor has five extracellular immunoglobulin-like domains and two intracellular tyrosine kinase domains. PDGFR chains are shown in *blue* (PDGFRα) and *red* (PDGFRβ). PDGF ligands are shown in *green* (PDGF-A), *yellow* (PDGF-B), *orange* (PDGF-C) and *purple* (PDGF-D).

Platelet-derived growth factors (PDGFs) acting via their tyrosine kinase receptors are major mitogens for many cell types of mesenchymal origin, including fibroblasts and vascular smooth muscle cells (VSMCs) [1-4]. Their role in enhancing migratory and proliferative responses and extracellular matrix (ECM) synthesis in these cells makes them key regulators of critical biological and pathological functions including tissue remodelling, scarring and fibrosis. Two PDGF receptor (PDGFR) isoforms (PDGFRα and PDGFRβ) form three different dimeric receptors – αα, ββ and αβ [5,6]. These receptors can interact with five different dimeric PDGF ligands: PDGF-AA, PDGF-BB, PDGF-CC, PDGF-DD and PDGF-AB [7-11], with different specificities and efficacies [12] (Figure 1).

While PDGF ligands have considerable overlap in their cellular signalling, individual ligands have also been found to control, or are dominant in, specific cellular events. PDGF-AA is a potent mitogen for cardiac fibroblasts and has been shown to be critical in lung alveolar myofibroblast development and alveogenesis [13,14]. PDGF-BB is required in the ontogeny of kidney mesangial cells and has been shown to be essential for development of the vasculature and vascular integrity [15]. PDGF-CC has been implicated in all phases of wound healing while blockade of PDGF-CC signalling inhibits pathological angiogenesis by acting on multiple cellular and molecular targets [16]. PDGF-DD is thought to stimulate angiogenesis and deposition of ECM and to be involved in hepatic and renal fibrosis [17]. It is also thought to be involved in VSMC phenotypic modulation and is upregulated in endothelial cells exposed to atherosclerosis-prone flow patterns [18].

*In vitro*, PDGFR isoforms have been shown to be potent activators of fibroblast proliferation, migration and survival [10]. Although stimulation of PDGFRα and PDGFRβ evokes similar signal transduction cascades, *in vitro* studies suggest distinct requirements for specific pathways to initiate particular receptor-mediated functions. For example, while activation of both receptors evoke mitogenic signals, stimulation of PDGFRα inhibits chemotaxis of fibroblasts and smooth muscle cells; in contrast, PDGFRβ activation potently stimulates fibroblast chemotaxis [9,19].

Recent studies have attempted to dissect PDGFR-specific events using genetically defined mouse embryonic fibroblasts (MEFS) expressing PDGFRα, PDGFRβ, both or neither [20]. These cells were generated by transducing PDGFRβ−/− cells with retroviral expression vectors for PDGFRα, PDGFRβ or both. Microarray gene expression array analysis provided some interesting insights. No genes were differentially expressed in the double null cells, suggesting minimal receptor-independent signalling. Whilst there is considerable overlap between PDGFRα and PDGFRβ signalling, this study identified transcripts that were differentially expressed between the cell lines. Thirty-three gene sets (functional groups of genes) were activated by PDGFRα only and 15 genes sets by PDGFRβ only. Interestingly, 25 genes sets were specifically activated by the heterodimeric receptors, for example, PDGFRα/β-activated components of the NFkB and interleukin (IL)-6 pathways, PDGFRα-activated C21-steroid hormone biosynthesis, and PDGFRβ activated the angiogenesis and epidermal growth factor receptor (EGFR) signalling pathways. The PDGFRα null cell line but not the PDGFRβ null or wild type (WT) showed differential expression of guanosine diphosphate (GDP) signalling genes [20]. Conversely, the differentially expressed gene sets particular to the PDGFRβ null and WT cell lines characterise ketosteroid metabolism [20]. Whilst these types of studies provide a reasonable genetic characterisation, they supply very little functional information, especially given that MEFs do not necessarily reflect the behaviour of adult fibroblasts.

Inhibition of both PDGFRs by broad-spectrum tyrosine kinase inhibitors such as Gleevec (which also inhibits c-Abl, c-kit and VEGFR) is used in the treatment of gastrointestinal stromal tumours and chronic mylogenous leukaemia [21-23]. They have been shown to reduce proliferation of normal mesangial cells via reduction in STAT3 phosphorylation [24] and of fibroblasts via reduction in PDGFRβ phosphorylation [25]. Gleevec treatment has also been shown to reduce the synthesis of ECM proteins in a model of dermal fibrosis [26]. These data suggest that PDGFR is regarded as a key molecular target in the development of anti-fibrotic therapies.

Taken together, these *in vitro* studies implicate PDGFR signalling in fibroblast function during tissue repair and scarring, however, questions still remain regarding the underlying mechanism(s) and specificity of PDGF ligand-receptor function. In this report, we used PDGFR-specific neutralising antibodies to block signalling through either PDGFRα or PDGFRβ to dissect out receptor-specific signalling events *in vitro*. We also analyse the role of the receptors on fibroblast migration and collagen gel contraction.

## Results

### Phosphorylation of PDGFR by PDGFAA, BB, CC and DD

**Figure 2:**
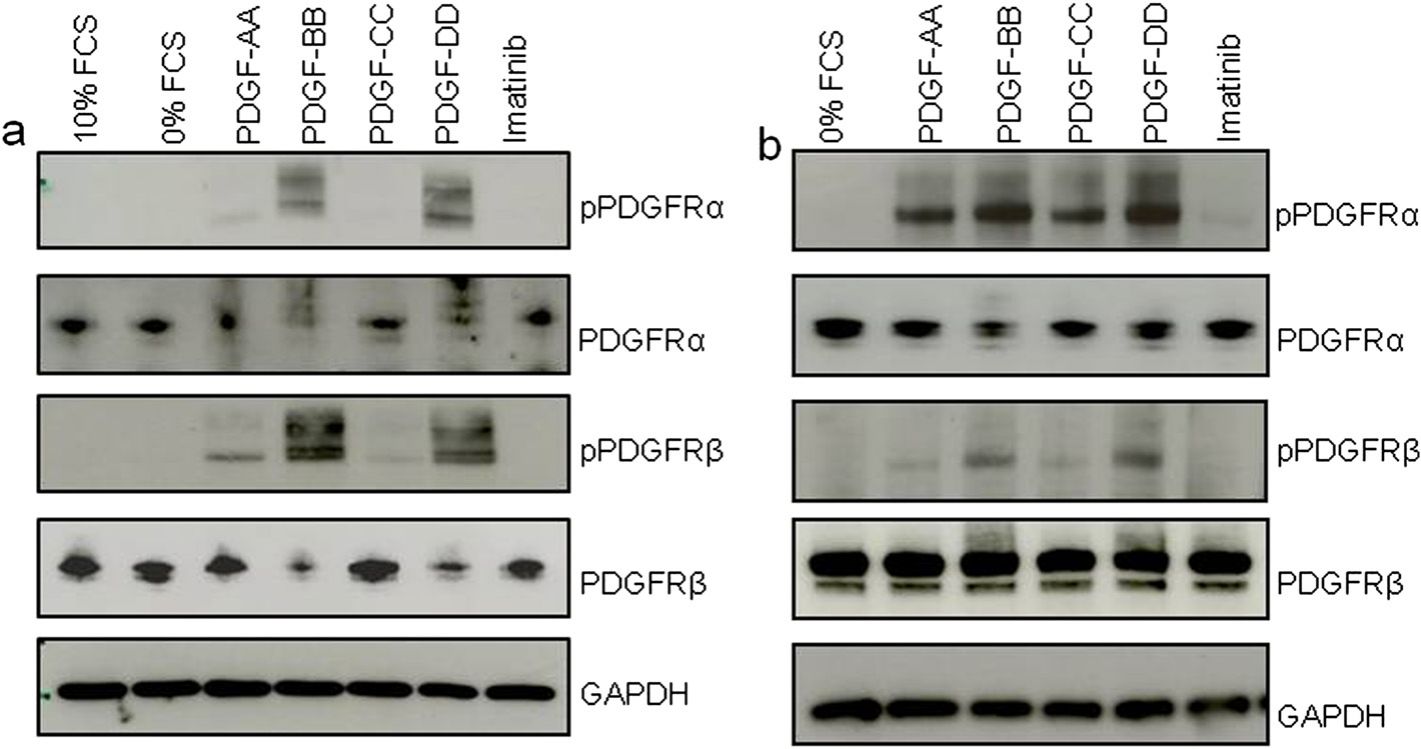
**Phosphorylation of PDGFRα and PDGFRβ in response to PDGF ligands.** Dermal fibroblasts (**a**) and lung fibroblasts (**b**) were grown in 10% FCS and serum-starved overnight or kept in 10% FCS. Cells were then stimulated with either 10% FCS, 0% FCS, PDGF-AA, PDGF-BB, PDGF-DD or Imatinib for 15 min. Whole cell lysate were Western blotted using antibodies against phospho-PDGFRα, PDGFRα, phospho-PDGFRβ, PDGFRβ and GAPDH (loading control).

In order to establish the pattern of phosphorylation of PDGFRs with the various PDGF ligands in human dermal fibroblasts, cells were serum-starved overnight and stimulated with PDGF-AA, PDGF-BB, PDGF-CC, PDGF-DD (20 ng/ml) or 10% FCS, or maintained in 0% FCS for 15 min. Western blots of total cell protein were probed with antibodies against phospho-PDGFRα, PDGFRα, phospho-PDGFRβ, PDGFRβ and GAPDH as a loading control (Figure 2a). Phosphorylation of PDGFRα was observed when cells were stimulated with PDGF-BB, PDGF-DD and, to a lesser extent, PDGF-AA. There was no detectable phosphorylation of PDGFRα when stimulated with PDGF-CC. Phosphorylation of PDGFRβ was observed after stimulation with PDGF-BB and PDGF-DD. A similar phosphorylation pattern of PDGFRβ is observed in lung fibroblasts when stimulated with PDGF-AA, PDGF-BB and PDGF-DD (Figure 2b). However, phosphorylation of PDGFRα was observed when cells were stimulated with all with PDGF ligands in lung fibroblasts.

**Figure 3:**
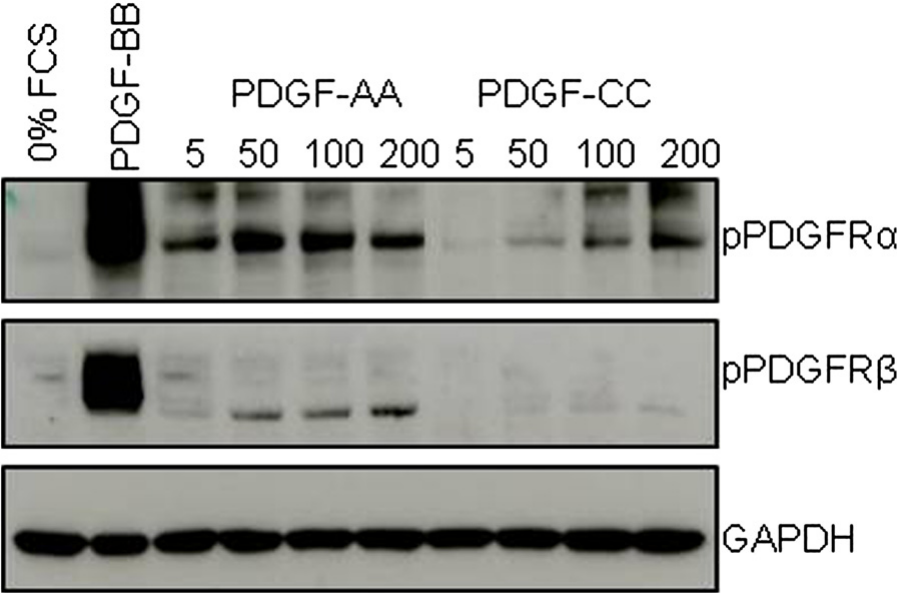
**Phosphorylation of PDGFRα and PDGFRβ in response different doses of PDGF-AA and PDGF-CC ligands.** Dermal fibroblasts were grown in 10% FCS and serum-starved overnight. Cells were stimulated with 0% FCS, PDGF-BB, PDGF-AA (5-200ng/ml) and PDGF-CC (5-200ng/ml) for 15 min. Whole cell lysates were Western blotted using antibodies against phospho-PDGFRα, phospho-PDGFRβ and GAPDH (loading control).

To ascertain if skin fibroblast PDGFRs were phosphorylated at higher doses of PDGF-CC and PDGF-AA, cells were stimulated with various concentrations of PDGF-AA or PDGF-CC (0–200 ng/ml). Western blots of total cell protein were probed with antibodies against phospho-PDGFRα, phospho-PDGFRβ and GAPDH as a loading control (Figure 3). Phosphorylation of PDGFRα was observed upon stimulation with PDGF-AA at doses 5–200 ng/ml. PDGFRα was phosphorylated moderately at 50 ng/ml and more strongly above 100 ng/ml by PDGF-CC. Phosphorylation of PDGFRβ was observed at a low level when stimulated with PDGF-AA at doses 50–200 ng/ml. No phosphorylation of PDGFRβ was detected after treatment with PDGF-CC.

### Effect of blocking antibody on phosphorylation and signal transduction

**Figure 4:**
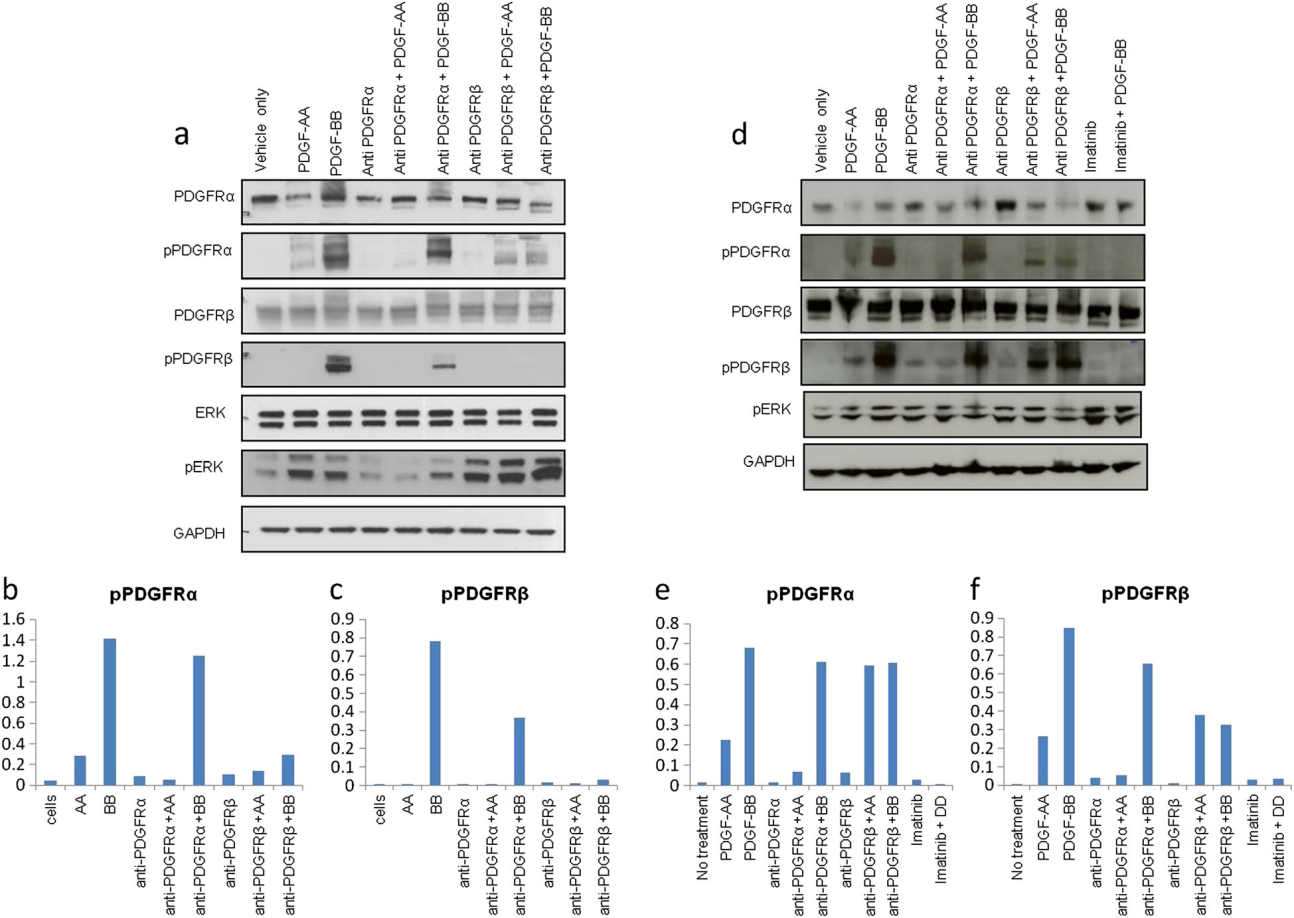
**Western blot showing effects of PDGFRα and PDGFRβ neutralising antibodies.** (**a**) Dermal fibroblasts were treated with neutralising antibodies to anit-PDGFRα, anti-PDGFRβ or vehicle for 1 h at room temperature and then stimulated with either PDGF-AA or PDGF-BB or vehicle for 15 min. Cells were then washed in ice-cold PBS and lysed. Cell lysates were Western blotted for PDGFRα, pPDGFRα, PDGFRβ, pPDGFRβ, ERK, pERK and GAPDH. The relative amount of pPDGFRα, pPDGFRβ, as measure by densitometry, is shown in (**b**) and (**c**). (**d**) Lung fibroblasts were treated and analysed in the same manner (**e**) and (**f**).

Human dermal fibroblasts were treated with PDGFRα or PDGFRβ neutralising antibodies and stimulated with PDGF-AA or PDGFR-BB ligands. Western blot analysis shows that the expression of total PDGFRα, PDGFRβ and ERK is similar across all treatment groups (Figure 4a). Phosphorylation of PDGFRα is observed when cells are stimulated with PDGF-AA or PDGF-BB alone and when treated with neutralising antibodies against PDGFRα and stimulated with PDGF-BB or with anti-PDGFRβ stimulated with either PDGF-AA or PDGF-BB. Phosphorylation of PDGFRα was not observed when cells were treated with either neutralising antibody alone or when treated with anti-PDGFRα and stimulated with the PDGFRα-specific ligand, PDGF-AA. Phosphorylation of PDGFRβ was observed when cells were stimulated with the universal PDGF ligand, PDGF-BB only and when treated with anti-PDGFRα and stimulated with PDGF-BB. Both receptors show enhanced phosphorylation in response to PDGF-BB compared to PDGF-AA (pPDGFRα 7-fold difference between PDGF-AA and PDGF-BB stimulation, PDGFRβ 700-fold difference between PDGF-AA and PDGF-BB stimulation) (Figure 4b-c). Phosphorylation of ERK is reduced in cells treated with anti-PDGFRα compared to controls (Figure 4a), but not when treated with anti-PDGFRβ. The PDGF receptor neutralising antibodies were also observed to block phosphorylation of their respective homodimer receptors in lung fibroblasts (Figure 4d-f). Phosphorylation of ERK was observed with most treatments.

### Effects of PDGFR neutralising antibodies on collagen gel contraction

**Figure 5:**
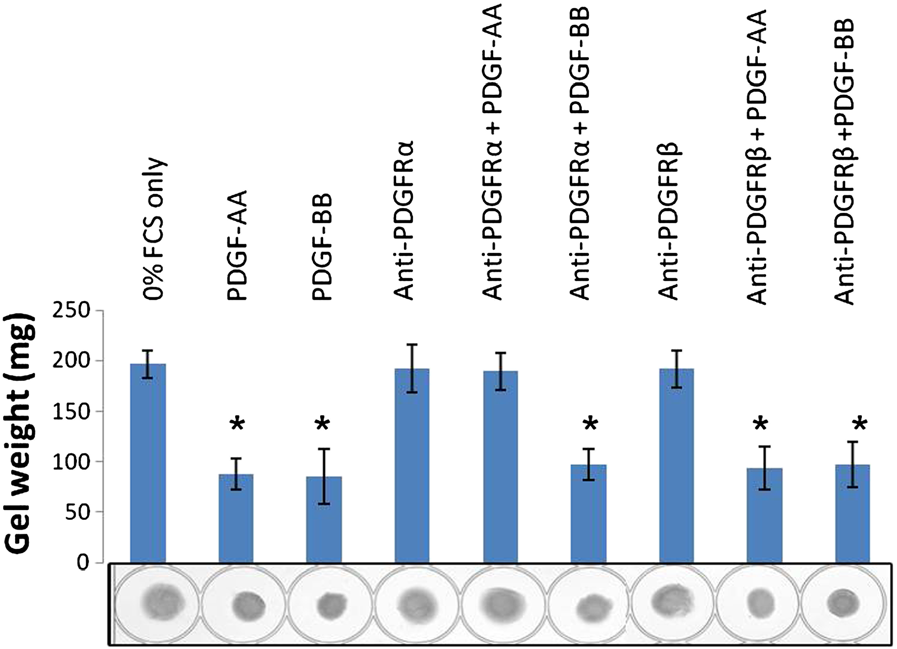
**The effect of collagen gel contraction of dermal fibroblasts after treatment with PDGFRα and PDGFRβ neutralising antibodies.** Dermal fibroblasts were treated with neutralising antibodies to anti-PDGFRα, anti-PDGFRβ or blank for 1 h at room temperature and were added to a collagen gel. The gels were then grown in media containing either PDGF-AA or PDGF-BB. After 24 h the gels were weighed to assess contraction. Error bars are standard error of the mean. Two-sample T-test statistical analysis was performed compared to compared gel weights compared to the 0% FCS control *p* = 0.004 PDGF-AA, *p* = 0.032 PDGF-BB, *p* = 0.82 anti-PDGFR-α., *p* = 0.62 anti-PDGFR-α.+ PDGF-AA, *p* = 0.03 anti-PDGFR-α.+ PDGF-BB, *p* = 0.77 anti-PDGFR-β, *p* = 0.035 anti-PDGFR-β + PDGF-AA, *p* = 0.0007 anti-PDGFR-β + PDGF-BB. *p > 0.05.

To analyse the effect of PDGFR neutralising antibodies on the ability of fibroblasts to contract collagen gels, dermal fibroblasts were treated with anti-PDGFR neutralising antibodies prior to embedding in collagen gels. The gels were then incubated in medium containing PDGF-AA, PDGF-BB or a 0% serum (control) for 24 h. The gel diameter was measured and gels weighed. Both PDGF-AA and PDGF-BB significantly induced collagen gel contraction in human dermal fibroblasts compared to 0% FCS (Figure 5) (*p* = 0.004 and *p* = 0.032 respectively). A similar effect was observed in gels where the cells were treated with anti-PDGFRα and stimulated with PDGF-BB (*p* = 0.031) or anti-PDGFRβ and stimulated with either PDGF-AA or PDGF-BB (*p* = 0.035 and *p* = 0.0007) compared to control. However, treatment with anti-PDGFRα completely blocked contraction induced by the PDGFRα-specific ligand PDGF-AA (*p* = 0.62). Treatment with either antibody alone had no effect on collagen gel contraction.

### Effect of PDGFR neutralising antibodies on fibroblast migration

**Figure 6:**
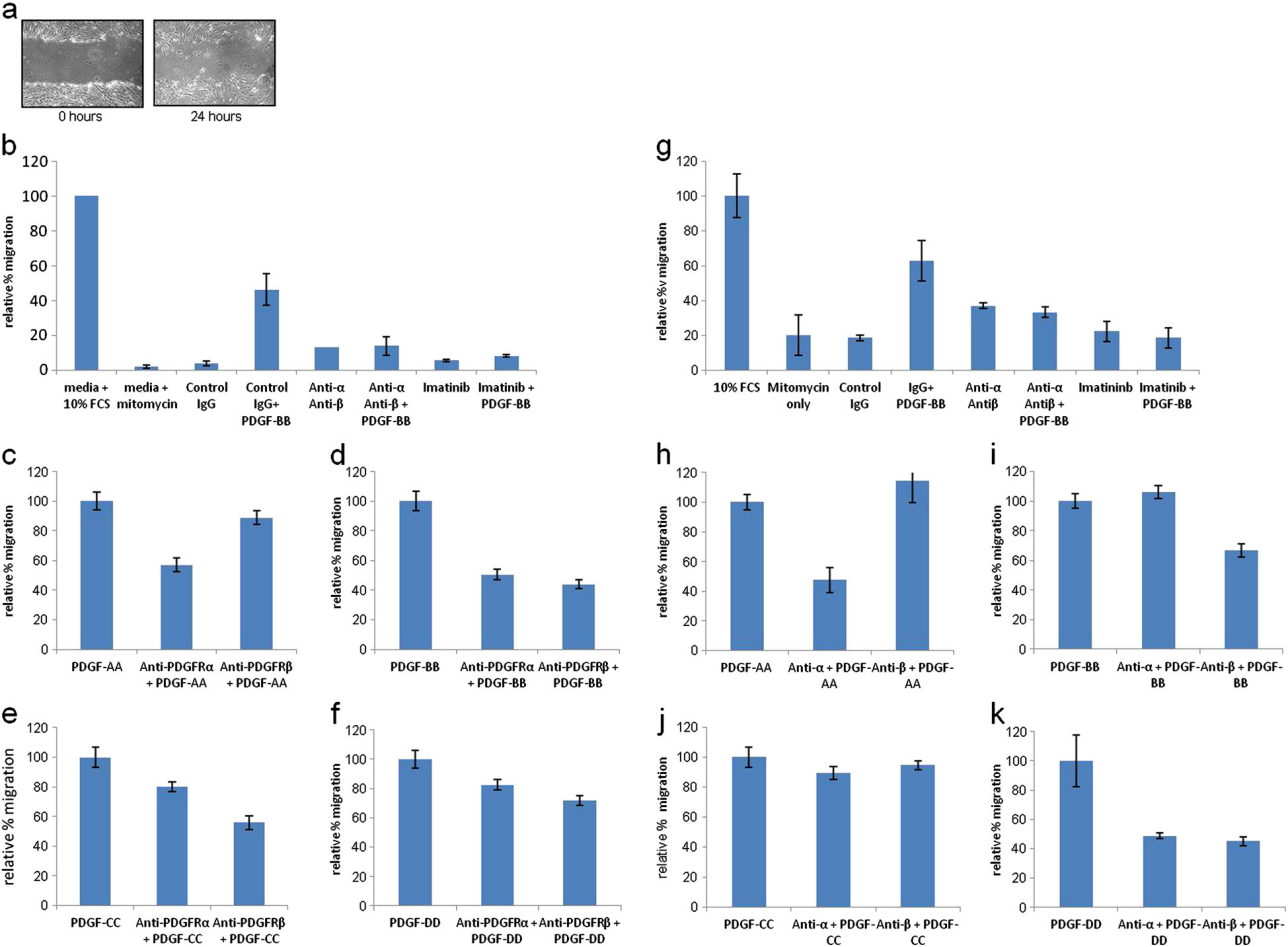
**The effect on migration of dermal and lung fibroblasts after treatment with PDGFRα and PDGFRβ neutralising antibodies.** Dermal fibroblasts were grown in 10% serum. A scratch was made in the cell layer and cells were treated with mitomycin C with neutralising antibodies to either anti-PDGFRα, anti-PDGFRβ, anti-PDGFRα and anti-PDGFRβ, control IgG, Imatinib or 10% FCS for 1 h at room temperature and then stimulated with PDGF-AA, PDGF-BB, PDGF-CC or PDGF-DD. (**a**). Percentage migration was calculated by measuring the average density of cells migrated into the scratched area after 24 h (**b**-**f**). *Error bars* are standard errors of the mean. Two-sample T-test was performed - 10% FCS vs. Media + mitomycin C, 10% FCS vs. IgG treated, anti-PDGFRα + anti-PDGFRβ vs. anti-PDGFRα + anti-PDGFRβ + PDGF-BB, Imatinib vs. Imatinib + PDGF-BB, *p* = 0.032, 0.035, 0.92_,_ and 0.1 respectively. Anti-PDGFRα + PDGF-AA vs. PDGF-AA, anti-PDGFRβ + PDGF-AA vs. PDGF-AA, anti-PDGFRβ + PDGF-BB vs. PDGF-BB, anti-PDGFRα + PDGF-BB vs. PDGF-BB, anti-PDGFRβ + PDGF-CC vs. PDGF-CC, anti-PDGFRα + PDGF-CC vs. PDGF-CC, anti-PDGFRβ + PDGF-DD vs. PDGF-DD, anti-PDGFRα + PDGF-DD vs. PDGF-DD —*p* = 0.24, 0.93, 0.006, 0.24, 0.06, 0.34, 0.38 and 0.64 respectively. Lung fibroblasts were treated and analysed in identical fashion. (**g**-**k**). Two-sample T-test was performed - FCS vs. Media + mitomycin C, 10% FCS vs. IgG treated, anti-PDGFRα + anti-PDGFRβ vs. anti-PDGFRα + anti-PDGFRβ + PDGF-BB, Imatinib vs. Imatinib + PDGF-BB —*p* = 0.585, 0.18, 0.64 and 0.21 respectively. Anti-PDGFRα + PDGF-AA vs. PDGF-AA, anti-PDGFRβ + PDGF-AA vs. PDGF-AA, anti-PDGFRβ + PDGF-BB vs. PDGF-BB, anti-PDGFRα + PDGF-BB vs. PDGF-BB, anti-PDGFRβ + PDGF-CC vs. PDGF-CC, anti-PDGFRα + PDGF-CC vs. PDGF-CC, anti-PDGFRβ + PDGF-DD vs. PDGF-DD, anti-PDGFRα + PDGF-DD vs. PDGF-DD —*p* = 0.25, 0.75, 0.05, 0.64, 0.82, 0.69, 0.1 and 0.23 respectively.

To investigate the effect PDGFR-neutralising antibodies on PDGF-mediated migration, a scratch wound assay was performed in dermal and lung fibroblasts. Cells were cultured in the presence of anti-proliferative agent, mitomycin C, treated with PDGFR-neutralising antibodies and stimulated with PDGF ligands. After 24 h, the mean density of cells in the scratched area was calculated and normalised against the migration induced by growth factor alone (Figure 6). Cells incubated in serum-free media containing only mitomycin C migrated the least compared to 10% FBS (*p* = 0.032 10% FCS vs. Media + mitomycin C) (Figure 6b). Similarly, a control IgG did not appear to have any effect on cell migration when used to pre-treat cells (*p* = 0.035 10% FCS vs. IgG treated); however, when stimulated with PDGF-BB, cells migrated to a greater extent (50% compared to 10% FCS). When cells were treated with both the anti-PDGFRα and anti-PDGFRβ neutralising antibodies or tyrosine kinase inhibitor, Imatinib, cell migration was reduced and did not increase significantly when stimulated with PDGF-BB (*p* = 0.92 anti-PDGFRα + anti-PDGFRβ vs. anti-PDGFRα + anti-PDGFRβ + PDGF-BB and *p* = 0.1 Imatinib vs. Imatinib + PDGF-BB) (Figure 6b). Whilst both PDGFR neutralising antibodies had an effect in reducing PDGF-induced migration, anti-PDGFRα had the greatest effect in abrogating PDGF-AA-stimulated migration compared to the ligand only control (55% migration) (*p* = 0.24 anti-PDGFRα + PDGF-AA vs. PDGF-AA, *p* = 0.93 anti-PDGFRβ + PDGF-AA vs. PDGF-AA) (Figure 6c). Anti-PDGFRβ was observed to have the greatest effect on reducing migration stimulated by PDGF-BB (45% compared to 50% when pre-incubated with anti-PDGFRα) (*p* = 0.006 anti-PDGFRβ + PDGF-BB vs. PDGF-BB, *p* = 0.24 anti-PDGFRα + PDGF-BB vs. PDGF-BB). Similarly, both PDGF-CC (55% compared to 80% when pre-incubated with anti-PDGFRα) and PDGF-DD-mediated cell migration were abrogated most effectively by treatment with anti-PDGFRβ (70% compared to 80% when pre-incubated with anti-PDGFRα) (*p* = 0.06 anti-PDGFRβ + PDGF-CC vs. PDGF-CC, *p* = 0.34 anti-PDGFRα + PDGF-CC vs. PDGF-, *p* = 0.38 anti-PDGFRβ + PDGF-DD vs. PDGF-DD, *p* = 0.64 anti-PDGFRα + PDGF-DD vs. PDGF-DD) (Figures 6d-f). This pattern is also observed in lung fibroblasts (Figure 6g-k). These data show that treating cells with neutralising antibodies against PDGFRα and PDGFRβ appears to slow the rate of cell migration and there is a synergistic inhibitory effect on the two antibodies.

## Discussion

Fibroblasts play a critical role in wound healing and tissue repair [2]. Signalling through the PDGF/PDGFR axis is a key feature of enhanced migration and ECM synthesis and are required for correct wound healing [1]. However, dysregulated activity and function of PDGFs are also believed to be important determinants of human diseases including excessive dermal scarring, many forms of organ-based tissue fibrosis as well as vascular diseases such as atherosclerosis and pulmonary hypertension [27]. It remains to be established whether a particular combination of PDGF/PDGFR is implicated in promoting certain disease pathologies.

The phosphorylation pattern of PDGFRα and PDGFRβ in response to PDGF-ligand stimulation observed in this study is similar to that previously reported [28-31]. Whilst it is firmly established that PDGF-AA is most specific for PDGFRα *in vitro*, reports vary as to whether phosphorylation of PDGFRs in response to PDGF-DD stimulation is PDGFRβ-specific or also stimulates PDGFRα [11,32]. Our studies indicated that in primary human dermal fibroblasts PDGF-DD stimulates PDGFRα and PDGFRβ to a similar extent, consistent with the findings of LaRochelle *et al.*[32]. However, a similar pattern of PDGFRα phosphorylation is not observed in lung fibroblasts. Phosphorylation of PDGFRα does not appear to have the same ligand specificity as dermal fibroblasts and was shown to be stimulated by all PDGF ligands. This tissue-specific difference in PDGFR stimulation is a previously unreported finding and a subject of further investigation.

As deletion of either PDGFR *in vivo* produces an embryonic lethal phenotype, it is difficult to assess the roles of the individual PDGF receptors [33-35]. Much of the previous *in vivo* work is therefore focused on the contribution of PDGFR in embryonic development. However, some conditional models also exist and work on these models is becoming increasingly prevalent [36].

Similarly, *in vitro* it has been difficult to dissect out receptor-specific signalling pathways as PDGFRs are reported to have redundancy and display compensatory effects [20]. Previous work by Wu *et al.* used specific PDGFR knockout cell lines created by generating MEFS from double knockout mice, then transducing retroviral PDGFRα or PDGFRβ vectors into the cells to express one or other of the proteins [20]. However, these analyses must be interpreted with caution as MEFS do not reflect adult fibroblast function and behaviour. Studies that examine the effect of specific point mutations of the PDGFRs in downstream signing pathways have been useful in dissecting receptor-specific cellular events [37-39]. However, they offer only limited insights in to the overall function of the receptor. Conversely, small-molecular inhibitors such as Gleevec are too broad in their range of target molecules to define PDGFR-specific cellular effects [22].

Ingram *et al.* have used PDGF-AA neutralising antibodies to great effect in the study of cytokine involvement in lung fibrosis [40]. In this report we investigate the use of PDGFR-specific neutralising antibodies in dissecting out PDGFRα- and PDGFRβ-specific events in functional assays migration and collagen gel remodelling.

We show that neutralising antibodies against PDGFRα and PDGFRβ block signalling through PDGFRα and PDGFRβ receptors as expected. The phosphorylation of each PDGFR receptor was reduced when cells were treated with specific anti-PDGFRα or anti-PDGFRβ neutralising antibodies. Similarly we have shown that the neutralising antibodies abrogate the signal from PDGFRα in specific downstream signalling cascades. This is best illustrated in the reduction of pERK in response to anti-PDGFRα neutralising antibodies in dermal fibroblasts (Figure 4). In order to determine the efficacy of both the neutralising antibodies, we previously analysed the phosphorylation of their PDGFR-α or PDGFR-β receptors using different antibodies raised against a number of different phosphorylation sites (PDGFR-α Y751 and Y1021, PDGFR-β Y1018 and Y754) (data not shown). In each case we found that both PDGFR-α and PDGFR-β were not activated and were henceforth satisfied that the neutralising antibodies had abrogated normal signal transduction through the receptors. However, stimulation of pERK was observed when treated with anti-PDGFRβ alone and in the presence of PDGF-AA and PDGF-BB ligands. Whilst Anti-PDGFRβ has been previously reported to bind the receptor at a site other than that of the ligand, and hence not stimulate the receptor in the conventional manner, it may however be acting as an auto-antibody. As phosphorylation of PDGFRβ was not observed in our measurements, it may be that the signal transduction pathway that mediates the phosphorylation of ERK may be acting through a different phosphorylation site on the PDGFRβ receptor. We have similarly observed a reduction in pAkt in the presence of anti-PDGFR neutralising antibodies compared to stimulation with PDGF-AA and PDGF-BB ligand in both skin and lung fibroblasts (data not shown). We do not observe significant activation of the receptor above the levels of the ligand alone in lung fibroblasts. The presence of auto-antibodies that stimulate PDGFRs has previously been reported by Baroni *et al.*[41]. We suggest that the neutralisation effect might be limited to actions mediated by specific phosphorylation sites on the PDGFRβ.

We also show that PDGFR-neutralising antibodies reduce PDGF-induced collagen gel remodelling and PDGF-mediated migration in a manner that reflects known, well-established receptor/ligand specificities (Figure 1). The role of PDGF in cell migration has long been established [4,11]. However, much of the current research has used the universal PDGF ligand (PDGF-BB) when examining the role of PDGF signalling in migration. The extent of dermal fibroblast migration under the various conditions investigated here is in accordance with the known receptor/ligand specificities. This supports the findings of Gao *et al.* who also showed that depletion of PDGFRβ in dermal fibroblasts results in decreased migration and therefore validates the use of neutralising antibodies as a method of dissecting PDGFR-specific events [36].

The anti-PDGFRα and anti-PDGFRβ antibodies bind at a different site on the receptors to that of the ligands and henceforth do not act in a competitive manner. As a consequence of this, it appears as though the neutralising antibodies do not block signalling through the PDGFRα/PDGFRβ heterodimer as phosphorylation of the PDGFRα receptor was still observed when cells were treated with anti-PDGFRα and stimulated with the universal PDGF ligand, PDGF-BB. This is difficult to prove for the PDGFRβ in the context of this study as there is no PDGFRβ-specific ligand. This would obviously have to be taken into consideration in any future studies as a potential caveat. However, this does enable specific investigation of signalling via PDGFR homodimers only.

## Conclusions

Similarly to other deletion strategies, the use of neutralisation antibodies has caveats associated with the extent and length of effect. However, this study has shown that PDGFRα and PDGFRβ neutralising antibodies can be a useful tool in studying PDGFR isoform-specific cellular events.

## Methods

### Cell culture

Human dermal and lung fibroblasts were isolated and cultured as previously described [42]. Cells were maintained in Dulbecco’s-modified Eagle’s medium (DMEM; Life Technologies Ltd., UK) supplemented with 10% foetal bovine serum (Life Technologies), 100 U/ml penicillin and 100 mg/ml streptomycin (Life Technologies) and cultured in a humidified atmosphere of 5% CO_2_. At confluence, cells were passaged 1:4 using trypsin-EDTA (Life Technologies).

### Treatment with neutralising antibodies

Cells were cultured in to 90% confluence in DMEM 10% FBS and serum starved (DMEM 0% FBS) overnight. Neutralising antibodies to either PDGFRα or PDGFRβ (R&D Systems, UK) were added (10 μg/ml - ND_50_ 1–6 μg/ml based on the manufacturer’s guidelines) to the cells in fresh serum-free media and incubated for 1 h at room temperature. Cells were then stimulated with vehicle, PDGF-AA, PDGF-DD, PDGF-CC (R&D Systems) or PDGF-BB (Abcam UK), at various concentrations (0–200 ng/ml) for either 15 min for Western blot analysis or 24 h for migration assays.

### Western blot analysis

Cell layers were washed in ice-cold PBS and lysed in RIPA buffer containing protease and phosphate inhibitors (Sigma UK). Equal amounts of protein (20 μg) were subjected to SDS/PAGE using 4–12% Bis Tris gels (Life Technologies). Proteins were blotted onto nitrocellulose as previously described [43], and proteins were detected using anti-PDGFRα, anti-PDGFRβ, anti-GAPDH (Abcam, UK), anti-phospho PDGFRα, anti-phospho PDGFRβ, (R&D Systems), anti-ERK and anti-phospho ERK antibodies followed by an appropriate HRP-conjugated secondary antibody (Cell Signalling, UK). Antibody binding was visualised Proteins were detected using an enhanced chemiluminescence kit (Amersham/GE Healthcare, UK).

### Migration assay

Cells were plated in 96-well plates and cultured in DMEM 10% FBS to 100% confluency, then serum-starved overnight. The cell layers were scratched using a 96 pin floating array (V and P Scientific, USA) and washed 2× in PBS. All media subsequently used were supplemented with mitomycin C (5 ng/ml; Sigma) to block cell proliferation. Cells were then treated with PDGFR neutralising antibodies as described above, then stimulated with PDGF-AA, PDGFR-BB, PDGF-CC, PDGF-DD (20 ng/ml) or vehicle for 24 h and imaged using an Olympus CK2 microscope (Olympus, UK) and Ziess axiocam MR camera (Carl Zeiss Ltd., UK). Mean density of cells that had migrated into the scratched area was calculated using Axio Vision software (Carl Zeiss Ltd.).

### Remodelling of collagen matrices

Twenty-four-well plates were coated with 2% bovine serum albumin (BSA) in PBS (2 ml/well) and incubated at 37°C overnight. The plates were then washed 3× with PBS. A collagen gel solution, consisting of one part 0.2 M N-2-hydroxyethylpiperazine-N’-2ethanesulphonic acid (HEPES), pH 8.0, four parts collagen [3 mg/ml, First Link (UK) Ltd., UK] and five parts DMEM was prepared. Cells were treated with neutralising antibodies against either PDGFRα or PDGFRβ (10 μg/ml) at room temperature for 1 h. A cell/collagen suspension was made, with a final concentration of 80,000 cells/ml and 1.2 mg/ml collagen. The cell/collagen suspension (1 ml per well) was added to the plates and incubated at 37°C to allow the collagen to polymerise. After 1 h, 1 ml of DMEM containing PDGF-AA, PDGFR-BB (20 ng/ml) or no serum control was gently added to each well resulting in detachment of the collagen gels from the tissue culture plastic. After 24 h, gels were measured and weighed as a measure of gel contraction [44].

## Abbreviations

BSA: Bovine serum albumin; ECM: Extracellular matrix; EGFR: Epidermal growth factor receptor; ERK: Extracellular signal-regulated kinase; GDP: Guanosine diphosphate; HEPES: N-2-hydroxyethylpiperazine-N’-2ethanesulphonic acid; NFκB: Nuclear factor kappa-light-chain-enhancer of activated B cells; PDGF: Platelet- derived growth factor; PDGFR: Platelet-derived growth factor receptor; pERK: Phospho extracellular signal-regulated kinase; VEGFR: Vascular epidermal growth factor receptor; VSMC: Vascular smooth muscle cell; WT: Wild type.

## Competing interests

None of the authors have any competing interests.

## Authors’ contributions

JD carried out the cell biology studies and drafting of the manuscript. XS harvested the original cell line and carried out the collagen gel contraction assay. JN participated in the design of the study and in drafting of the manuscript. DA participated in the design of the study and in drafting of the manuscript. All authors read and approved the final manuscript.

## References

[B1] Alvarez RH Kantarjian HM Cortes JE Biology of platelet-derived growth factor and its involvement in disease Mayo Clin Proc 2006 81 9 1241 57 10.4065/81.9.1241 16970222

[B2] Andrae J Gallini R Betsholtz C Role of platelet-derived growth factors in physiology and medicine Genes Dev 2008 22 10 1276 312 10.1101/gad.1653708 18483217 PMC2732412

[B3] Grotendorst GR Platelet-derived growth factor is a chemoattractant for vascular smooth muscle cells J Cell Physiol 1982 113 2 261 6 10.1002/jcp.1041130213 6184376

[B4] Seppa H Platelet-derived growth factor in chemotactic for fibroblasts J Cell Biol 1982 92 2 584 8 10.1083/jcb.92.2.584 7061598 PMC2112065

[B5] Claesson-Welsh L Ronnstrand L Heldin CH Biosynthesis and intracellular transport of the receptor for platelet-derived growth factor Proc Natl Acad Sci U S A 1987 84 24 8796 800 10.1073/pnas.84.24.8796 2827155 PMC299637

[B6] Heldin CH Ostman A Ronnstrand L Signal transduction via platelet-derived growth factor receptors Biochim Biophys Acta 1998 1378 1 F79 113 9739761 10.1016/s0304-419x(98)00015-8

[B7] Raines EW Ross R Platelet-derived growth factor. I. High yield purification and evidence for multiple forms J Biol Chem 1982 257 9 5154 60 7068680

[B8] Deuel TF Human platelet-derived growth factor. Purification and resolution into two active protein fractions J Biol Chem 1981 256 17 8896 9 7263691

[B9] Heldin CH Wasteson A Westermark B Platelet-derived growth factor Mol Cell Endocrinol 1985 39 3 169 87 10.1016/0303-7207(85)90061-9 2984061

[B10] Li X PDGF-C is a new protease-activated ligand for the PDGF alpha-receptor Nat Cell Biol 2000 2 5 302 9 10.1038/35010579 10806482

[B11] Bergsten E PDGF-D is a specific, protease-activated ligand for the PDGF beta-receptor Nat Cell Biol 2001 3 5 512 6 10.1038/35074588 11331881

[B12] Donovan J Abraham DA Norman J Platelet-derived growth factor signalling in mesenchymal cells Frontiers in Biosciences 2012 10.2741/4090 23276912

[B13] Simm A Nestler M Hoppe V PDGF-AA, a potent mitogen for cardiac fibroblasts from adult rats J Mol Cell Cardiol 1997 29 1 357 68 10.1006/jmcc.1996.0280 9040050

[B14] Bostrom H PDGF-A signaling is a critical event in lung alveolar myofibroblast development and alveogenesis Cell 1996 85 6 863 73 10.1016/S0092-8674(00)81270-2 8681381

[B15] Hellstrom M Role of PDGF-B and PDGFR-beta in recruitment of vascular smooth muscle cells and pericytes during embryonic blood vessel formation in the mouse Development 1999 126 14 3047 55 10375497 10.1242/dev.126.14.3047

[B16] Hou X PDGF-CC blockade inhibits pathological angiogenesis by acting on multiple cellular and molecular targets Proc Natl Acad Sci U S A 2010 107 27 12216 21 10.1073/pnas.1004143107 20566880 PMC2901428

[B17] Kumar A Platelet-derived growth factor-DD targeting arrests pathological angiogenesis by modulating glycogen synthase kinase-3beta phosphorylation J Biol Chem 2010 285 20 15500 10 10.1074/jbc.M110.113787 20231273 PMC2865282

[B18] Thomas JA PDGF-DD, a novel mediator of smooth muscle cell phenotypic modulation, is upregulated in endothelial cells exposed to atherosclerosis-prone flow patterns Am J Physiol Heart Circ Physiol 2009 296 2 H442 52 19028801 10.1152/ajpheart.00165.2008 PMC2643880

[B19] Heldin CH Westermark B Mechanism of action and in vivo role of platelet-derived growth factor Physiol Rev 1999 79 4 1283 316 10508235 10.1152/physrev.1999.79.4.1283

[B20] Wu E Comprehensive dissection of PDGF-PDGFR signaling pathways in PDGFR genetically defined cells PLoS One 2008 3 11 e3794 10.1371/journal.pone.0003794 19030102 PMC2582946

[B21] Heinrich MC Inhibition of c-kit receptor tyrosine kinase activity by STI 571, a selective tyrosine kinase inhibitor Blood 2000 96 3 925 32 10910906

[B22] Buchdunger E Abl protein-tyrosine kinase inhibitor STI571 inhibits in vitro signal transduction mediated by c-kit and platelet-derived growth factor receptors J Pharmacol Exp Ther 2000 295 1 139 45 10991971

[B23] Buchdunger E O'Reilly T Wood J Pharmacology of imatinib (STI571) Eur J Cancer 2002 38 Suppl 5 S28 36 12528770 10.1016/s0959-8049(02)80600-1

[B24] Hirai T PDGF receptor tyrosine kinase inhibitor suppresses mesangial cell proliferation involving STAT3 activation Clin Exp Immunol 2006 144 2 353 61 10.1111/j.1365-2249.2006.03073.x 16634810 PMC1809660

[B25] Soria A The effect of imatinib (Glivec) on scleroderma and normal dermal fibroblasts: a preclinical study Dermatology 2008 216 2 109 17 10.1159/000111507 18216472

[B26] Distler JH Imatinib mesylate reduces production of extracellular matrix and prevents development of experimental dermal fibrosis Arthritis Rheum 2007 56 1 311 22 10.1002/art.22314 17195235

[B27] Perros F Platelet-derived growth factor expression and function in idiopathic pulmonary arterial hypertension Am J Respir Crit Care Med 2008 178 1 81 8 10.1164/rccm.200707-1037OC 18420966

[B28] Hammacher A Isoform-specific induction of actin reorganization by platelet-derived growth factor suggests that the functionally active receptor is a dimer EMBO J 1989 8 9 2489 95 2479551 10.1002/j.1460-2075.1989.tb08385.x PMC401239

[B29] Seifert RA Two different subunits associate to create isoform-specific platelet-derived growth factor receptors J Biol Chem 1989 264 15 8771 8 2542288

[B30] Hart CE Two classes of PDGF receptor recognize different isoforms of PDGF Science 1988 240 4858 1529 31 10.1126/science.2836952 2836952

[B31] Fredriksson L Li H Eriksson U The PDGF family: four gene products form five dimeric isoforms Cytokine Growth Factor Rev 2004 15 4 197 204 10.1016/j.cytogfr.2004.03.007 15207811

[B32] LaRochelle WJ PDGF-D, a new protease-activated growth factor Nat Cell Biol 2001 3 5 517 21 10.1038/35074593 11331882

[B33] Soriano P Abnormal kidney development and hematological disorders in PDGF beta-receptor mutant mice Genes Dev 1994 8 16 1888 96 10.1101/gad.8.16.1888 7958864

[B34] Soriano P The PDGF alpha receptor is required for neural crest cell development and for normal patterning of the somites Development 1997 124 14 2691 700 9226440 10.1242/dev.124.14.2691

[B35] Orr-Urtreger A Developmental expression of the alpha receptor for platelet-derived growth factor, which is deleted in the embryonic lethal Patch mutation Development 1992 115 1 289 303 1322271 10.1242/dev.115.1.289

[B36] Gao Z Deletion of the PDGFR-beta gene affects key fibroblast functions important for wound healing J Biol Chem 2005 280 10 9375 89 15590688 10.1074/jbc.M413081200

[B37] Ronnstrand L Identification of two C-terminal autophosphorylation sites in the PDGF beta-receptor: involvement in the interaction with phospholipase C-gamma EMBO J 1992 11 11 3911 9 1396585 10.1002/j.1460-2075.1992.tb05484.x PMC556901

[B38] Yokote K Structural determinants in the platelet-derived growth factor alpha-receptor implicated in modulation of chemotaxis J Biol Chem 1996 271 9 5101 11 10.1074/jbc.271.9.5101 8617789

[B39] Yu JC Tyrosine mutations within the alpha platelet-derived growth factor receptor kinase insert domain abrogate receptor-associated phosphatidylinositol-3 kinase activity without affecting mitogenic or chemotactic signal transduction Mol Cell Biol 1991 11 7 3780 5 1646396 10.1128/mcb.11.7.3780-3785.1991 PMC361148

[B40] Ingram JL IL-13 and IL-1beta promote lung fibroblast growth through coordinated up-regulation of PDGF-AA and PDGF-Ralpha FASEB J 2004 18 10 1132 4 15155567 10.1096/fj.03-1492fje

[B41] Baroni SS Stimulatory autoantibodies to the PDGF receptor in systemic sclerosis N Engl J Med 2006 354 25 2667 76 10.1056/NEJMoa052955 16790699

[B42] Abraham DJ Tumor necrosis factor alpha suppresses the induction of connective tissue growth factor by transforming growth factor-beta in normal and scleroderma fibroblasts J Biol Chem 2000 275 20 15220 5 10.1074/jbc.275.20.15220 10809757

[B43] Shi-Wen X Endogenous endothelin-1 signaling contributes to type I collagen and CCN2 overexpression in fibrotic fibroblasts Matrix Biol 2007 26 8 625 32 10.1016/j.matbio.2007.06.003 17681742

[B44] Dooley A Modulation of collagen type I, fibronectin and dermal fibroblast function and activity, in systemic sclerosis by the antioxidant epigallocatechin-3-gallate Rheumatology (Oxford) 2010 49 11 2024 36 10.1093/rheumatology/keq208 20627968

